# Association between serum lipid and all-cause mortality in asthmatic populations: a cohort study

**DOI:** 10.1186/s12944-024-02179-w

**Published:** 2024-06-21

**Authors:** Jun Wen, Rongjuan Zhuang, Qingliu He, Chengcheng Wei, Mohan Giri, Jing Chi

**Affiliations:** 1 Department of Respiratory and Critical Care Medicine, The First Affiliated Hospital of Chongqing Medical University, Chongqing Medical University, Chongqing, China; 2 Department of Urology, The Second Affiliated Hospital of Fujian Medical University, Quanzhou, China; 3 Department of Urology, Tongji Medical College, Union Hospital, Huazhong University of Science and Technology, Wuhan, Hubei China; 4 Department of Urology, The First Affiliated Hospital of Chongqing Medical University, Chongqing, China

**Keywords:** Lipid, Low-density lipoprotein-cholesterol (LDL-C), Mortality, Asthma, National Health and Nutrition Examination Survey (NHANES); CoxBoost

## Abstract

**Background:**

Presently, the majority of investigations primarily evaluate the association between lipid profiles and asthma. However, few investigations explore the connection between lipids and mortality related to the disease. This study aims to explore the association of serum lipids with all-cause mortality within asthmatic adults.

**Methods:**

The investigation included 3233 eligible patients with asthma from the NHANES (2011–2018). The potential associations were explored using three Cox proportional hazards models, restricted cubic splines (RCS), threshold effect models, and CoxBoost models. In addition, subgroup analyses were conducted to investigate these associations within distinct populations.

**Results:**

After controlling all covariables, the Cox proportional hazards model proved a 17% decrease in the probability of death for each increased unit of low-density lipoprotein-cholesterol (LDL-C) (mmol/L). Yet, there was no association seen between blood high-density lipoprotein cholesterol (HDL-C), total cholesterol, or triglyceride and all-cause mortality in asthmatics. The application of RCS and threshold effect models verified an inverse and linear association of LDL-C with all-cause mortality. According to the results from the CoxBoost model, LDL-C exhibited the most substantial impact on the follow-up status of asthmatics among the serum lipids.

**Conclusion:**

Our investigation concluded that in American asthmatic populations, LDL-C levels were inversely and linearly correlated with mortality. However, no independent relationship was found between triglycerides, total cholesterol, or HDL-C and mortality.

**Supplementary Information:**

The online version contains supplementary material available at 10.1186/s12944-024-02179-w.

## Introduction

Globally, asthma affects about 300 million individuals and is a widespread chronic respiratory illness with high morbidity and mortality [1]. It is a diverse illness that causes bronchial hyperresponsiveness, reversible airflow obstruction, and inflammation. Those who have it may experience symptoms including tightness in the chest, dyspnea, and wheezing [2, 3].

As the body of high-caliber research on asthma has grown, so too has our understanding of the disease’s risk factors, underlying mechanisms, and therapeutic therapy [4]. The possible link between asthma and metabolic illnesses has been the subject of an increasing number of studies in recent years [5, 6]. There is increasing evidence from laboratory and clinical studies showing that the lipid mediators are closely related to the onset and progression of asthma [7–9]. Asthma is a heterogeneous disease of complex etiology involving many types of cells [10]. Lipids and fatty acids are important for various cellular functions, during inflammation, hypoxic responses and immune responses in asthma [11]. For example, arachidonic acid (AA), the major lipid metabolite generated in the lungs of asthmatics, can contribute to asthma development by inducing T cell secretion, degranulation of eosinophils, and IgE generation by B cells [11]. However, extracellular eicosapentaenoic acid (EPA) and docosahexaenoic acid (DHA) can in turn alleviate bronchial inflammation by decreasing macrophage production of inflammatory factors such as tumor necrosis factor-αand interleukin 1β [12]. Not only that, apolipoproteins, lipoprotein particles, and lipids are thought to be significant modulators of inflammation [13, 14]. A sizable cross-sectional study that included 85,555 participants revealed a correlation between lipid alterations and wheezing symptoms in adults after controlling for obesity [15]. The development of asthma may be impacted by lipid metabolism’s apparent links to long-term inflammation and bronchial remodeling in both large and small airways [7, 9].

Moreover, a growing number studies have found that lipid levels are associated with mortality in the general population [16] and in certain diseases [17–19] during long-term follow up. Nevertheless, there aren’t many comprehensive investigations on the connection between lipid profiles and prognosis in asthmatics, and this relationship has gotten less attention. The National Health and Nutrition Examination Survey (NHANES) data were the subject of a secondary analysis by us. The aim of this investigation was to discuss the association of lipid profiles, including cholesterol, triglyceride, high density lipoprotein-cholesterol (HDL-C), and low density lipoprotein-cholesterol (LDL-C) levels, with all-cause mortality in asthmatic patients in America.

## Materials and methods

### Study data and population

**Fig. 1:**
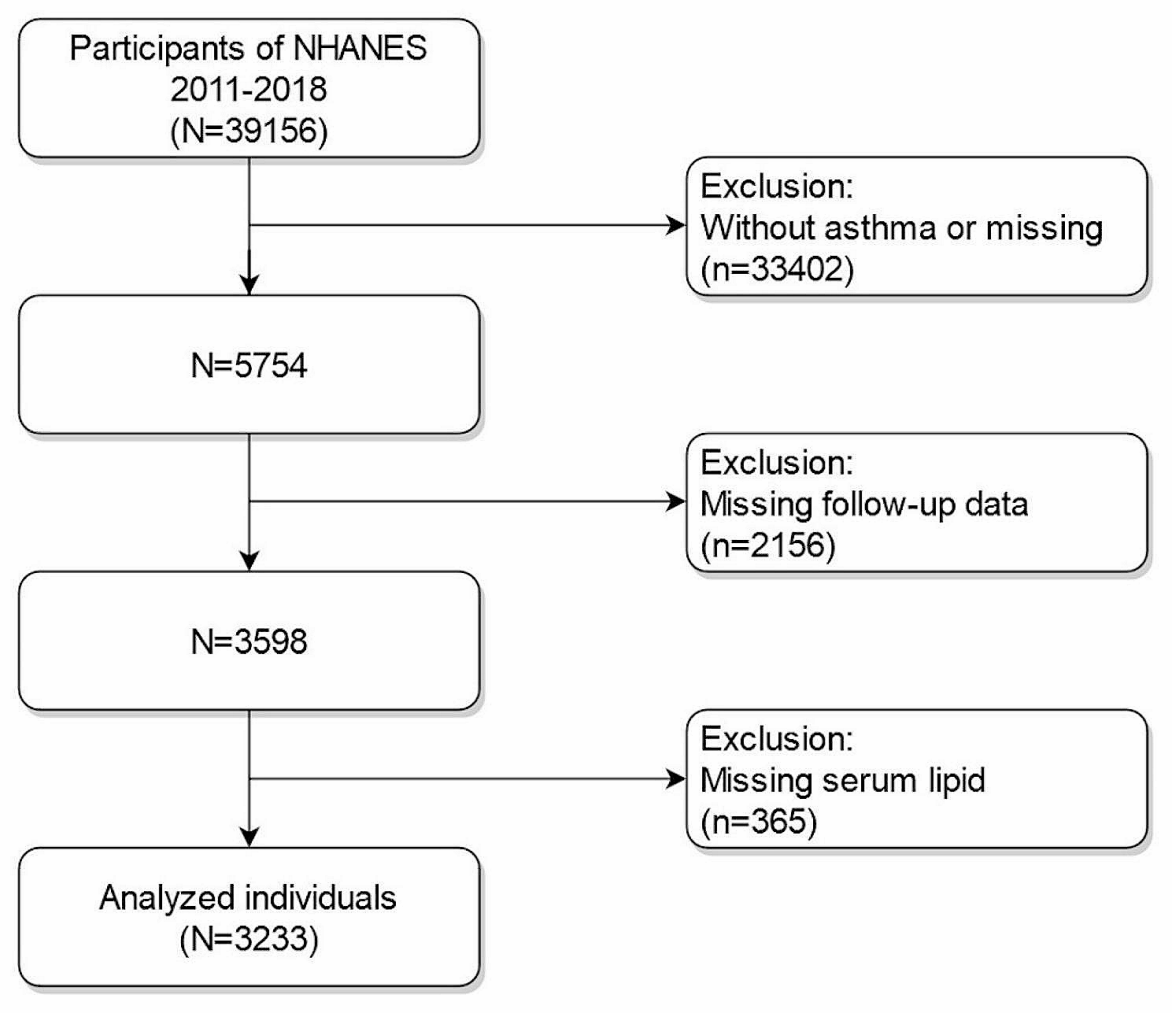
Flow diagram of selecting populations for analysis

The Centers for Disease Control and Prevention (CDC) carried out the NHANES, a vital scientific endeavor that methodically evaluates the health and nutritional condition of both American populations. Figure 1 illustrates that NHANES had a participation of 39,156 people from 2011 to 2018. Adhering to specific inclusion and exclusion criteria, our study population excluded: (1) individuals without asthma (*n* = 31,812) or missing asthma diagnosis (*n* = 1590); (2) those with missing survival status data (*n* = 2156); (3) those lacking serum lipid data (*n* = 365). Asthma status was ascertained using standardized questionnaires presented during participants’ visits, employing the query, “Have you ever been diagnosed with asthma by a physician or other healthcare professional?” Participants who answered positively were classified as having a diagnosis of asthma. Finally, our investigation involved a huge sample of 3233 asthmatic people in the USA.

### Assessment of exposure and outcome variables

Serum lipids were the main exposure variables in our research. Using enzymatic assays, cholesterol, triglyceride, HDL-C, and LDL-C levels were determined. And we employed unique study identifiers and performed probabilistic matching with the National Death Index (NDI) as of December 31, 2018, to ascertain the vital status of our participants. The NCHS provided additional information on the matching methodology. In addition, we used the 10th edition of the International Statistical Classification of Diseases (ICD) to determine mortality status. We primarily focused on all-cause mortality. Detailed information can be obtained at the https://www.cdc.gov/nchs/data-linkage/mortality-public.htm.

### Covariables

In order to address the potential confounding effects of diverse factors, we incorporated numerous covariables into our investigation. The covariables considered in the analysis were sex, age, race, education, poverty-to-income ratio (PIR), marriage, body mass index (BMI) (kg/m2), waist circumference (cm), smoking status (smoker: person with a history of smoking over 100 cigarettes; non-smoker: person with a history of smoking less than 100 cigarettes), intake of alcohol intake (gm), total fat intake (gm), hypertension history, diabetes history, cardiovascular disease (CVD) history, chronic obstructive pulmonary disease (COPD) history, depression history, lipid-lowering drug (whether lipid-lowering drugs used in past a month), glucocorticoid drug (whether glucocorticoid drugs used in past a month), serum creatinine (umol/L), glycohemoglobin (%), white blood cell number (WBC) (1000 cells/uL), blood neutrophils number (BNEU) (1000 cells/uL), and blood eosinophils number (BEOS) (1000 cells/uL).

### Statistical analysis

Statistical analyses were conducted by R software (version 4.2.0). *P* < 0.05 was regarded as statistical significance. Initially, we developed three univariate and multivariate Cox proportional hazards regression models to assess the independent association of serum lipid with all-cause mortality in individuals with asthma. Three models were constructed, which were as follows: Model X (adjusted for none), Model Y (adjusted for age, race, sex, education, marriage, and PIR), and Model Z (adjusted for sex, age, race, education, marriage, PIR, BMI, waist circumference, smoking, alcohol intake, total fat intake, hypertension history, diabetes history, CVD history, COPD history, depression, lipid-lowering drug, glucocorticoid drug, serum creatinine, glycohemoglobin, WBC, BNEU, and BEOS). Subsequently, we utilized the trend test, restricted cubic spline (RCS), and threshold effect model to examine whether the association was linear or not. Then, we first conducted interaction tests on different populations and conducted subgroup analysis on populations with interactions. And CoxBoost algorithm model was employed to thoroughly estimate the relative effect size of serum lipid on the follow-up status. Finally, a Kaplan-Meier survival analysis was performed in order to assess the prognostic impact of LDL-C on populations with asthma. Multiple imputation (MICE package) was used to handle variables with missing values, and the specific number of missing variables was as follows: education (*n* = 4), marriage (*n* = 216), PIR (*n* = 264), BMI (41), waist circumference (*n* = 173), smoking (*n* = 58), alcohol intake (*n* = 237), total fat intake (*n* = 237), hypertension (*n* = 3), diabetes (*n* = 4), COPD (*n* = 222), glycohemoglobin (*n* = 4), WBC (*n* = 8), BNEU (*n* = 12), and BEOS (*n* = 12). And the missing covariates mentioned above were all random missing.

## Results

### Baseline characteristics based on survival status of study individuals

Provided in Table 1 were the baseline characteristics of people involved in the cohort study (*N* = 3233), grouped according to their follow-up outcomes. The individuals with asthma under analysis displayed an average age of 45.91 years, with 57.47% being female. The median period of follow-up for all samples was 57.93 months. And the number of survivors was 3044, and the number of deaths was 189. Significant variations were observed in the distributions of sex, age, race, education, marriage, PIR, waist circumference, smoking, fat intake, history of hypertension, diabetes, CVD, COPD and depression, usage of lipid-lowering and glucocorticoid drugs, serum creatinine, glycohemoglobin, WBC, BNEU, cholesterol, and LDL-C.

**Table 1:** The study population’s baseline characteristics based on the survival status

	Survival group (*N* = 3044)	Dead group (*N* = 189)	*P* value
Sex (%)			0.02
Male	42.02%	50.79%	
Female	57.98%	49.21%	
Age (years)	44.67 ± 18.14	65.94 ± 15.01	< 0.01
Race (%)			< 0.01
Non-Hispanic White	39.62%	55.02%	
Non-Hispanic Black	25.79%	24.87%	
Other Race	34.59%	20.11%	
Education (%)			< 0.01
Under high school	20.30%	30.69%	
High school	22.14%	23.81%	
Over high school	57.56%	45.50%	
Marriage status (%)			< 0.01
Married	45.93%	34.92%	
Single	45.89%	58.73%	
Living with a partner	8.18%	6.35%	
PIR	1.81 (0.93–3.69)	1.29 (0.82–2.08)	< 0.01
BMI (kg/m2)	30.76 ± 8.36	30.78 ± 8.80	0.98
Waist circumference (cm)	101.95 ± 18.81	105.21 ± 18.38	0.021
Smoking status (%)			< 0.01
Smoker	44.61%	64.02%	
Non-smoker	55.39%	35.98%	
Alcohol intake (gm)	9.26 ± 28.41	7.62 ± 25.24	0.44
Total fat intake (gm)	71.35 (43.85-103.19)	59.86 (34.90-92.07)	< 0.01
Hypertension (%)			< 0.01
No	61.86%	25.93%	
Yes	38.14%	74.07%	
Diabetes (%)			< 0.01
No	85.48%	62.96%	
Yes	14.52%	37.04%	
CVD history (%)			< 0.01
No	86.50%	54.50%	
Yes	13.50%	45.50%	
COPD history (%)			< 0.01
No	90.57%	66.14%	
Yes	9.43%	33.86%	
Depression history (%)			< 0.01
No	85.35%	74.07%	
Yes	14.65%	25.93%	
Lipid-lowering drugs (%)			< 0.01
No	86.14%	70.90%	
Yes	13.86%	29.10%	
Glucocorticoid drugs (%)			< 0.01
No	84.69%	64.02%	
Yes	15.31%	35.98%	
Serum creatinine (umol/l)	73.37 (62.76–87.52)	86.63 (69.84-112.27)	< 0.01
Glycohemoglobin (%)	5.74 ± 1.07	6.35 ± 1.46	< 0.01
WBC (1000 cells/uL)	7.47 ± 2.43	8.06 ± 3.64	< 0.01
BNEU (1000 cell/uL)	4.39 ± 1.82	5.05 ± 2.17	< 0.01
BEOS (1000 cells/uL)	0.20 (0.10–0.30)	0.20 (0.10–0.30)	0.08
Serum triglyceride (mmol/l)	1.26 (0.86–1.99)	1.34 (0.96–1.91)	0.65
Serum cholesterol (mmol/l)	4.84 ± 1.11	4.67 ± 1.27	0.04
Serum HDL-C (mmol/l)	1.37 ± 0.41	1.40 ± 0.64	0.41
Serum LDL-C (mmol/l)	3.12 ± 0.97	2.94 ± 1.03	0.01

Note: Continuous variables with a normal distribution are represented by the mean ± SE, while continuous variables with a non-normal distribution are represented by the median and IQR.Categorical variables were expressed as proportions

### Association between serum lipids and all-cause mortality in asthmatics

Associations between serum lipids and all-cause mortality in asthmatics were displayed in Table 2. The univariable and multivariable Cox proportional hazard models (Table 2) showed that higher LDL-C was associated with lower all-cause mortality in asthmatics. Yet, there were no statistically significant associations between triglyceride, cholesterol, HDL-C, and all-cause mortality in Model Z (adjusting for all covariables). In Model Z, it was proved that a 17% decrease in the probability of death for each increased unit of LDL-C (mmol/L). However, we discovered that only cholesterol had an independent negative association with all-cause mortality in non-asthmatic populations (Supplementary Table 1). In addition, the trend test (Table 3) indicated a possible nonlinear association of LDL-C with all-cause mortality in Model Z (*p* for trend > 0.05).

**Table 2:** Association between serum lipid and all-cause mortality in asthmatic adults

		Model X HR (95% CI) *P* value		Model Y HR (95% CI) *P* value		Model Z HR (95% CI) *P* value	
Serum LDL-C		0.79 (0.68, 0.93) < 0.01		0.76 (0.65, 0.89) < 0.01		0.83 (0.70, 0.97) 0.02	
Serum HDL-C		1.18 (0.85, 1.64) 0.32		1.08 (0.76, 1.55) 0.65		1.23 (0.80, 1.90) 0.35	
Serum cholesterol		0.86 (0.75, 0.99) 0.03		0.85 (0.74, 0.97) 0.02		0.88 (0.75, 1.03) 0.12	
Serum triglyceride		1.01 (0.94, 1.09) 0.73		0.99 (0.88, 1.10) 0.79		0.88 (0.75, 1.03) 0.11	

Note: Model X controlled for none. Model Y controlled for age, race, gender, education, marriage, and PIR. Model Z: Model Y + controlled for BMI, waist circumference, smoking, alcohol intake, total fat intake, hypertension, diabetes, CVD, COPD, depression, lipid-lowering drug, glucocorticoid drug, serum creatinine, glycohemoglobin, WBC, BNEU, and BEOS.

**Table 3:** Association between LDL-C and all-cause mortality in asthmatics

	Model X HR (95% CI) *P* value		Model Y HR (95% CI) *P* value		Model Z HR (95% CI) *P* value		
LDL-C	0.79 (0.68, 0.93) < 0.01		0.76 (0.65, 0.89) < 0.01		0.83 (0.70, 0.97) 0.02		
LDL-C tertiles groups							
T1 (0.575–2.613)	Reference		Reference		Reference		
T2 (2.614–3.430)	0.65 (0.46, 0.91) 0.01		0.65 (0.46, 0.92) 0.01		0.78 (0.55, 1.13) 0.19		
T3 (3.432–9.745)	0.61 (0.43, 0.86) 0.01		0.59 (0.42, 0.84) < 0.01		0.70 (0.48, 1.03) 0.07		
P for trend	< 0.01		< 0.01		0.07		

Note: Model X adjusted for none. Model Y adjusted for age, race, gender, education, marriage, and PIR. Model Y + controlled for BMI, waist circumference, smoking, alcohol intake, total fat intake, hypertension, diabetes, CVD, COPD, depression, lipid-lowering drug, glucocorticoid drug, serum creatinine, glycohemoglobin, WBC, BNEU, and BEOS.

### Restricted cubic splines (RCS) and threshold effect model

**Fig. 2:**
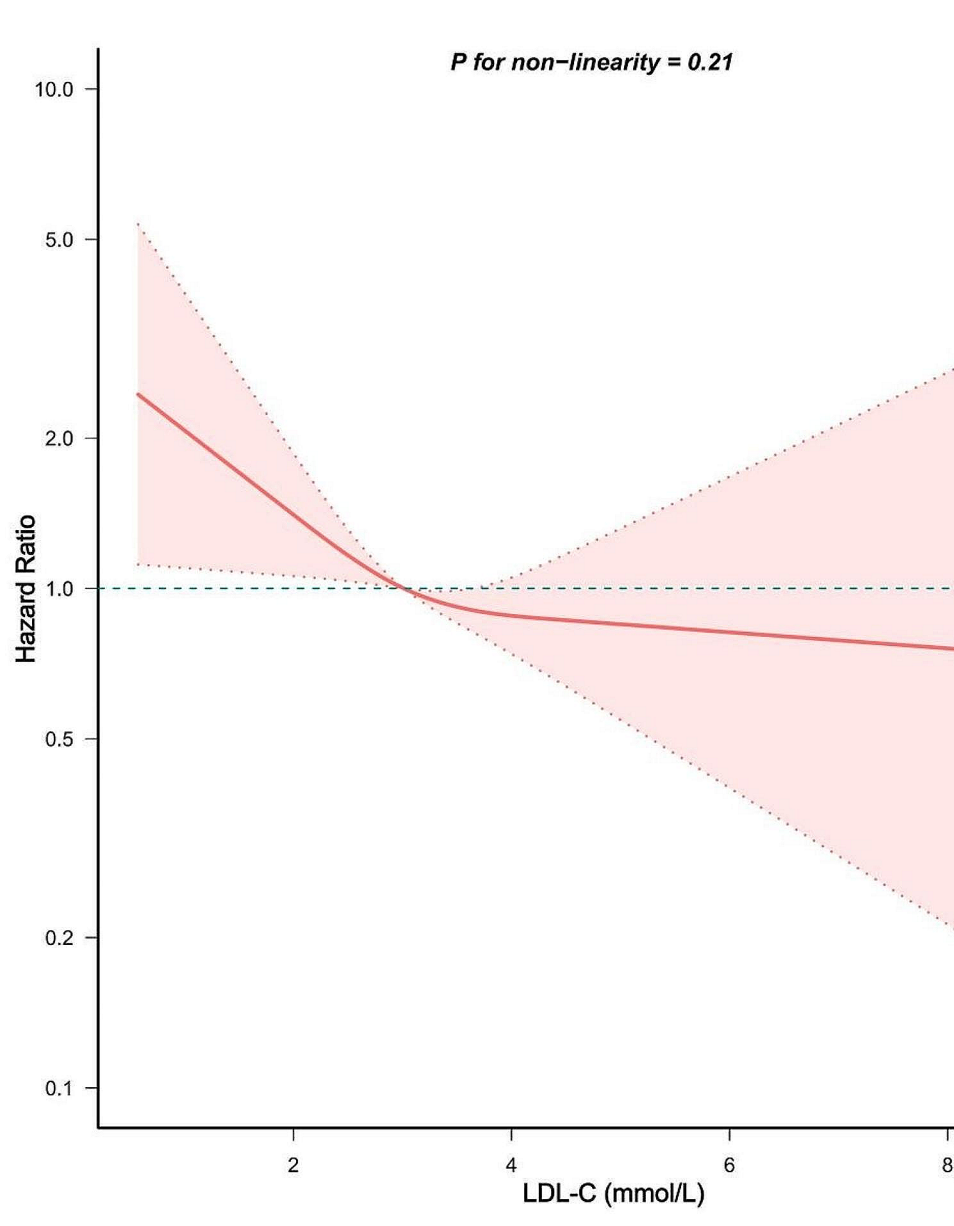
Association of serum LDL-C with all-cause mortality in asthmatics. The red solid line and red area correspond to the HR and their corresponding 95%CI, separately

Our investigation utilized RCS and a threshold effect model to verify whether the association of LDL-C with all-cause mortality in asthmatics was linear or not. In Fig. 2, the RCS, adjusting for all covariables, demonstrated an analysis of a linear and inverse association of LDL-C with all-cause mortality in asthmatics (P for non-linearity > 0.05). It was then shown by a threshold effect model that the link of LDL-C with all-cause mortality did not have a statistically significant inflection point (P for log-likelihood ratio > 0.05). Therefore, the one-line model showed more appropriateness in depicting the connection of LDL-C with all-cause mortality (Table 4). The aforementioned studies demonstrated a linearly inverse association of LDL-C with all-cause mortality in asthmatics.

**Table 4:** Threshold effect analysis of LDL-C and all-cause mortality in asthmatics

	HR (95% CI) *P* value
**Model 1**	
Linear effect	0.83 (0.70, 0.97) 0.02
**Model 2**	
Inflection point (K)	2.42
< K	0.52 (0.30, 0.91) 0.02
> K	0.92 (0.75, 1.12) 0.40
P for log likelihood ratio	0.10

Note: Model 1 and 2 all adjusted age, race, gender, education, marriage, PIR, BMI, waist circumference, smoking, alcohol intake, total fat intake, hypertension, diabetes, CVD, COPD, depression, lipid-lowering drug, glucocorticoid drug, serum creatinine, glycohemoglobin, WBC, BNEU, and BEOS.

### Subgroup analysis

Subgroup analyses and interaction tests were carried out to evaluate the associations of LDL-C with all-cause mortality throughout various populations (Supplementary Table 2). However, we only discovered an interaction between hypertension, LDL-C, and all-cause mortality (P for interaction < 0.05), and no interaction was found in other subgroups. Then, we ran a subgroup analysis based on a history of hypertension and discovered an inverse association of LDL-C with all-cause mortality in the population without hypertension.

### CoxBoost model

**Fig. 3:**
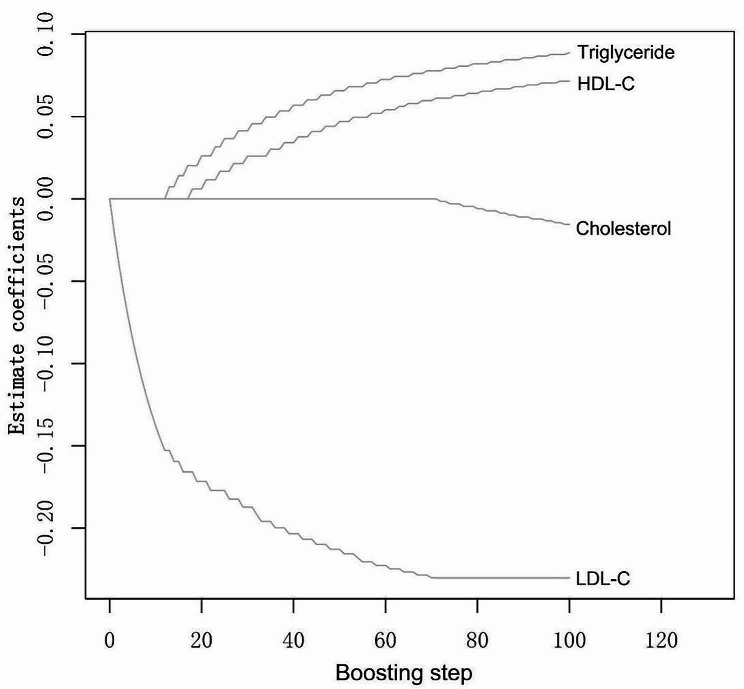
The CoxBoost model assessed the positive and negative effects size of serum lipids in relation to the follow-up status

To estimate the relative effect size of serum lipids on the follow-up status of the study populations, we employed the CoxBoost algorithm model (Fig. 3). This model evaluated the positive and negative effects of cholesterol, triglyceride, HDL-C, and LDL-C on all-cause mortality in asthmatics. The outcome of the CoxBoost model, illustrated in Fig. 3, revealed that triglycerides and HDL-C had a promoting impact on mortality risk. Conversely, serum lipids associated with a decreased risk of death, in descending order of effect size, were LDL-C and cholesterol. Among the serum lipids, LDL-C exhibited the most substantial impact on the follow-up status of asthmatics.

### Kaplan–Meier survival analysis

**Fig. 4:**
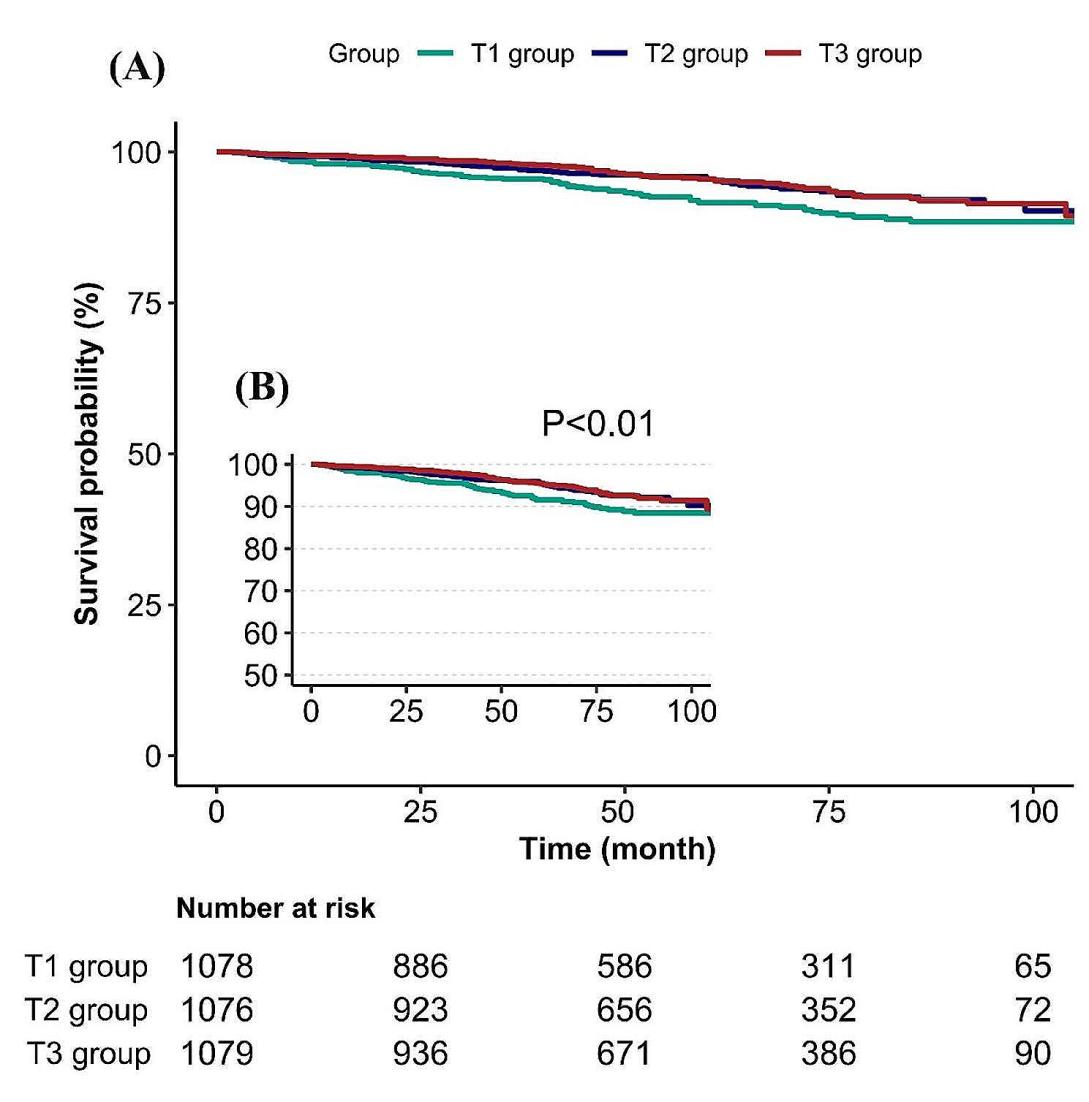
(**A**) Kaplan-Meier survival analysis for asthamtic populations based on LDL-C of tertile groups. (**B**) Figure B was a thumbnail of Figure A.T1-T3: LDL-C is grouped by tertiles

To evaluate the prognostic impact of LDL-C (Fig. 4A and B) on asthamtic populations, a Kaplan-Meier analysis was conducted. Kaplan-Meier survival analysis demonstrated that asthmatic people with high LDL-C (T2 and T3) exhibited an increased survival probability compared to those with low LDL-C (T1) (*p* < 0.01).

## Discussion

Our investigation represented the first longitudinal investigation to date that explored the association of serum lipids with prognosis in asthmatics. After controlling all covariables, the outcome proved that a 17% decrease in the probability of death for each increased unit of LDL-C (mmol/L). Yet, there were no independent associations of triglyceride, cholesterol, or HDL-C, with all-cause mortality in asthmatics. The RCS and threshold effect models further verified an inverse and linear association of LDL-C with all-cause mortality. And we discovered an interaction between hypertension, LDL-C, and all-cause mortality, and an inverse association of LDL-C with all-cause mortality in the population without hypertension. The CoxBoost model suggested LDL-C exhibited the most substantial impact on the follow-up status of asthmatics among serum lipids. And Kaplan-Meier survival analysis demonstrated that asthmatic people with high LDL-C exhibited an increased survival probability compared to those with low LDL-C.

We observed an independent association between serum LDL-C levels and all-cause mortality in the asthmatic population, but not in the non-asthmatic population (Supplementary Table 1). Several large-sample studies have explored the relationship between LDL-C levels and all-cause mortality in long-term follow-up, but the results are controversial. This association was U-shaped in the Danish general population [16], L-shaped in general adults enrolled in the NHANES in the United States [20], and negative in geriatric population [21–23]. It is now generally accepted that lowering LDL-C levels improves the prognosis of CVD. However, an observational study found that patients with the highest LDL-C levels after myocardial infarction (MI) have the highest risk of recurrent MI but the lowest risk of hospitalization for non-MI causes and mortality [24]. LCL-C variability has also been found to be associated with all-cause mortality in patients with type 2 diabetes [18]. A negative association of LDL-C levels with mortality has also been determined in Koreans who do not take lipid-lowering medications [25].

Although LDL-C has received substantial attention in the prediction of mortality risk, its molecular mechanisms remain unclear. Most current studies support the hypothesis that LDL-C levels are reduced by frailty and various diseases. Another explanation is that LDL may be capable of inactivating bacteria and viruses [26]. LDL neutralizes toxins released by microorganisms and protects cells from damage, which is a terrific natural anti-infection substance [27]. Lipids serve as potent signalling molecules, orchestrating a range of cellular responses and playing a significant role between different cells [28, 29]. It has been shown that LDL induces the anti-inflammatory cytokine leukotropin 10 and counteracts the inflammatory effects of lipopolysaccharide (LPS) [30].

LDL itself is beneficial because the body needs it to transport cholesterol synthesized by the liver to surrounding tissues to help repair cells, bind to bacterial toxins and defend against microbes [31]. However, if LDL contains large amounts of unsaturated fatty acids, it can be easily oxidized by free radicals to oxidized low-density lipoprotein (oxLDL). The smaller volume of oxLDL is unable to properly transport cholesterol but could induce foam cell formation and inflammatory responses [32]. Scichilone et al. distributed the LDL in serum into seven bands according to serum concentrations of LDL subclasses [33]. Larger LDL-1 and LDL-2 were defined as the least pro-inflammatory LDLs, and smaller LDL-3 to 7 were defined as the most pro-inflammatory LDLs [33]. Based on their findings, it can be inferred that the least pro-inflammatory LDL subclasses were positively correlated with lung function, while the most pro-inflammatory LDL subclasses were negatively correlated with lung function. In addition, a large cross-sectional study based on the United Kingdom Biobank (UKB) was carried out, using Mendelian randomised analysis, and found that asthma was also inversely correlated to TG, LDL, and TC, but was positively correlated to HDL [34]. However, Barochia’s results showed that LDL-C level is associated with airflow obstruction in idiopathic asthma [35]. These observations led us to explore the role of different subclasses of LDL in asthma. LDL-C might be an important mediator of inflammatory changes in the airways.

The results of the current studies strongly suggest a connection between lipid metabolism and asthma, although it remains unclear to date. Nevertheless, the prevalence of asthma is still growing in all countries [36, 37]. In adults, the frequency of asthma ranges from 4 to 10% [36, 38]. Asthma exacerbations result in 1.8 million hospitalizations per year, with an estimated mortality rate of 13.3 deaths per million per year in American adults [39]. Therefore, more prospective studies and basic experiments are necessary to further investigate this connection and the potential mechanisms.

Yet, we must acknowledge certain limitations in our investigation. We selected patients with asthma based on questionnaire responses rather than pulmonary function testing due to limitations in the database. And this study lacks information on the use of lipid-lowering drugs in the population during follow-up. Furthermore, there were unquantified variables in our analysis, and the existence of undiscovered confounding effects from these factors cannot be excluded. Moreover, the findings of this research were derived exclusively from the analysis of observational data and did not provide a direct illustration of the mechanism through which LDL-C impacts asthma mortality. Further research is required to investigate the biological connection between lipids and asthma mortality in order to identify possible treatment targets.

## Conclusion

The investigation proved a linear and inverse relationship between LDL-C levels and mortality in American individuals with asthma. However, there was no direct relationship found between serum HDL-C, total cholesterol, or triglyceride levels and mortality. In the future, more studies specifically targeted at investigating the regulation mechanisms of LDL-C in asthma and the connection between dyslipidemia and asthma are required.

### Electronic supplementary material

Below is the link to the electronic supplementary material.

Supplementary Material 1

## Data Availability

All accessible data is available on the official NHANES website (http://www.cdc.gov/nchs/nhanes/index.htm).
