# Supplementary material for: Association between serum lipid and all-cause mortality in asthmatic populations: a cohort study
Source: Lipids Health Dis. 2024 Jun 21;23:189. doi: 10.1186/s12944-024-02179-w (PMC11191228; doi:10.1186/s12944-024-02179-w)
Supplement: Supplementary file 1 — Supplementary Material 1 [file 12944_2024_2179_MOESM1_ESM.docx]

| **Supplementary Table 1.** Association between serum lipid and all-cause mortality in nonasthmatic adults. | | | |
| --- | --- | --- | --- |
|  | Model X | Model Y | Model Z |
|  | HR (95% CI) P value | HR (95% CI) P value | HR (95% CI) P value |
| Serum LDL-C | 0.79 (0.74, 0.84) <0.01 | 0.88 (0.83, 0.94) <0.01 | 0.94 (0.88, 1.00) 0.06 |
| Serum HDL-C | 0.88 (0.75, 1.02) 0.09 | 0.76 (0.65, 0.89) <0.01 | 0.84 (0.71, 1.00) 0.05 |
| Serum cholesterol | 0.80 (0.75, 0.85) <0.01 | 0.87 (0.82, 0.92) <0.01 | 0.92 (0.87, 0.98) 0.01 |
| Serum triglyceride | 1.01 (0.97, 1.05) 0.77 | 0.99 (0.94, 1.04) 0.76 | 0.96 (0.91, 1.02) 0.20 |
| **Note:** Model X controlled for none. Model Y controlled for age, race, gender, education, marriage, and PIR. Model Z: Model Y+ controlled for BMI, waist circumference, smoking, alcohol intake, total fat intake, hypertension, diabetes, CVD, COPD, depression, lipid-lowering drug, glucocorticoid drug, serum creatinine, glycohemoglobin, WBC, BNEU, and BEOS. | | | |

| **Supplementary Table 2.** Subgroup analysis and interactions between LDL-C and all-cause mortality in asthmatics. | | | |
| --- | --- | --- | --- |
| **Subgroup** | **N** | **HR (95% CI) P value** | **P for interaction** |
| **Sex** |  |  | 0.544 |
| Male | 1375 | **-** |  |
| Female | 1858 | - |  |
| **Age** |  |  | 0.09 |
| <40 | 1354 | - |  |
| 40-60 | 935 | - |  |
| ≥60 | 944 | **-** |  |
| **Race** |  |  | 0.10 |
| Non-Hispanic White | 1310 | - |  |
| Non-Hispanic Black | 832 | - |  |
| Other Race | 1091 | **-** |  |
| **Hypertension** |  |  | **0.04** |
| No | 1932 | **0.60 (0.42-0.86) 0.01** |  |
| Yes | 1301 | 0.90 (0.76-1.08) 0.27 |  |
| **Diabetes** |  |  | 0.26 |
| No | 2721 | - |  |
| Yes | 512 | **-** |  |
| **CVD history** |  |  | 0.17 |
| No | 2736 | - |  |
| Yes | 497 | **-** |  |
| **COPD history** |  |  | 0.60 |
| No | 2882 | - |  |
| Yes | 351 | - |  |
| **Depression history** |  |  | 0.59 |
| No | 2738 | - |  |
| Yes | 495 | - |  |
| **Glucocorticoid drugs** |  |  | 0.47 |
| No | 2699 | - |  |
| Yes | 534 | - |  |
| **Lipid-lowering drugs** |  |  | 0.30 |
| No | 2756 | **-** |  |
| Yes | 477 | - |  |
| Note: All stratified analyses adjusted for all covariates except for the stratification variable. | | | |
